# Tae-Eum Type as an Independent Risk Factor for Obstructive Sleep Apnea

**DOI:** 10.1155/2013/910382

**Published:** 2013-03-12

**Authors:** Seung Ku Lee, Dae Wui Yoon, Hyeryeon Yi, Si Woo Lee, Jong Yeol Kim, Chol Shin

**Affiliations:** ^1^Institute of Human Genomic Study, College of Medicine, Korea University Ansan Hospital, 516 Gojan-1-dong, Danwon-gu, Ansan 425-707, Republic of Korea; ^2^Department of Nursing, Korea Nazarene University, Wolbongro 48, Seobuk-gu, Cheonan 331-718, Republic of Korea; ^3^Constitutional Medicine and Diagnosis Research Group, Korea Institute of Oriental Medicine, 461-24 Jeonmin-dong, Yuseong-gu, Daejeon 305-811, Republic of Korea; ^4^Department of Pulmonary, Sleep and Critical Care Medicine, College of Medicine, Korea University Ansan Hospital, 516 Gojan-1-dong, Danwon-gu, Ansan 425-707, Republic of Korea

## Abstract

Obstructive sleep apnea (OSA) is prevalent and associated with several kinds of chronic diseases. There has been evidence that a specific type of Sasang constitution is a risk factor for metabolic and cardiovascular diseases that can be found in patients with OSA, but there are no studies that address the association between the Sasang constitution type (SCT) and OSA. The purpose of this study was to investigate the association between the SCT and OSA. A total of 652 participants were included. All participants were examined for demographic information, medical history, and completed an interviewer-administered questionnaire on life style and sleep-related variables. Biochemical analyses were performed to determine the glucose and lipid profiles. An objective recording of OSA was done with an unattended home PSG using an Embla portable device. The apnea-hypopnea index (AHI) and oxygen desaturation index (ODI) were significantly higher in the Tae-eum (TE) type as compared to the So-eum (SE) and the So-yang (SY) types. Even after adjusting for confounding variables, the TE type still had a 2.34-fold (95% CI, 1.11–4.94; *P* = 0.0262) increased risk for OSA. This population-based cohort study found that the TE constitutional type is an independent risk factor for the development of OSA.

## 1. Introduction

Sasang constitutional medicine (SCM) is a branch of Korean traditional medicine and regarded as a growing field of alternative and complementary medicine. Based on the basic concept that different types of constitutions have different susceptibilities to disease and drug treatments [1], SCM has classified human beings into four constitutions. The four constitutions of Tae-yang (TY), TE, SY, and SE types are based on physical traits such as body shape, external appearance, voice, psychological traits (temperament), and differences of functional activity among internal organ systems [1, 2].

According to the traditional SCM theory, each SCT represents different temperaments, body shapes, and functional activity of the four viscera: lungs, liver, spleen, and kidneys. Typically, a person with a TY type is creative, positive, heroic, and has a more developed lung area and a less developed liver area. The TE type refers to a cautious and endurable personality, and people with this type have a more developed liver area and a less developed lung area. The SE type can be characterized by an inactive and self-directed personality, and people characterized by this type typically have more developed kidney area and a less developed spleen area. The SY type has temperament of sharp, easily gets bored, and, unlike the SE type, is characterized by developed spleen area and a less developed kidney area. Each SCT also represents differently developed appearances: developed nape area in the TY type; developed chest in the SY type; developed waist in the TE type; developed hip in the SE type. Moreover, based on the previous studies that examined physical traits of SCT, the TE type has relatively elevated levels of body fat mass, blood pressure, and blood lipid such as total cholesterol, triglyceride, and low density lipoprotein as compared with the SY and SE types [2]. Researchers not only have been attempting to develop methods which can objectively and reliably classify constitution types but also have been investigating the relationship between Sasang constitution types (SCTs) and various kinds of pathologic conditions. As a result of the efforts, over the past decade, there has been a growing body of evidence revealing that the SCT may be independently associated with different types of chronic diseases. 

It has been reported that individual SCT has different blood glucose levels, high-density lipoprotein (HDL) cholesterol, blood pressure (BP), serum triglycerides (TG), and abdominal obesity, which are well-known risk factors for metabolic syndrome (MS) [3]. The TE and SY types especially have a higher risk for MS as compared to the SE type. In addition, it has been found that the four SCTs have different prevalence of diabetes mellitus (DM) [4] and hypertension [5] and may be function as risk factors for the diseases. This suggests that the appropriate classification of patients according to SCTs may helpful in predicting susceptibility to such kind of diseases and in prescribing efficient drugs, without any significant side effect. 

Obstructive sleep apnea (OSA) is a very common public health problem having a prevalence that ranges from 17% to 28% worldwide [6–8] and is a major cause of sleep disturbances. In Korea, the prevalence of OSA was 27.1% and 16.8% in men and women, respectively, when OSA was defined as an apnea-hypopnea index (AHI) as ≥5 [9]. Such results are similar to those of other populations including Caucasians [10]. 

OSA is characterized by repeated partial or complete obstruction of the upper airway during sleep. It accompanies intermittent hypoxemia and hypercapnia, repeated arousals, and an intrathoracic negative pressure swing [11, 12]. The physiological alterations shown in OSA patients result in an increase in the reactive oxygen species [13], the sympathetic nervous system activation [14, 15], and as inflammatory response elevation [16, 17], which are considered as the main biological mediators accounting for pathological conditions associated with OSA [18]. OSA is closely related with excessive daytime sleepiness [19], contributing to an increase in traffic accident rates [20, 21] and daytime dysfunction [22, 23], cardio-cerebrovascular morbidity [24–27], cognitive impairments [28], and insulin resistance [29, 30], which can raise socioeconomic cost and thus increases the public health burden. 

Although the OSA may independently contribute to the development of MS, DM, and hypertension, and the SCTs, especially the TE type, can act as a risk factor for the diseases, there is a lack of research findings that have reported a relationship between them. This study aimed, therefore, to investigate the relationship between SCTs and OSA in a large population-based cohort study. 

## 2. Materials and Methods

### 2.1. Subjects

We conducted a cross-sectional investigation using data from the Korean Genome and Epidemiology Study (KoGES). Briefly, members of the study cohort consisted of 5,020 Korean citizens (2,523 male and 2,497 female) aged from 40 to 69 years who participated from 2001 in a comprehensive health examination and on-site interview at Korea University Ansan Hospital. In the study, all participants participated in a comprehensive health examination including an anthropometric evaluation and completed an interviewer-administered questionnaire on demographic information, medical history, and health conditions. The cohort members were followed up biennially with a scheduled site visit for similar interviews and health examinations. Among 1,824 individuals who were enrolled in the cohort from 2010 to 2011 and were classified as SCT using an integrated diagnostic model developed by Do et al. [31], 652 individuals who completed portable PSG monitoring were selected for the analysis. Consistent with the previous finding that the TY type is extremely low in the population [4, 32], there was no one from our sample who was classified as the TY type. Therefore, only the data from TE, SE, and SY constitutions was evaluated in this study. 

### 2.2. Classification of SCT

Each constitutional type was determined by an integrated diagnostic model developed with a multinomial logistic regression based on four individual quantitative data areas such as facial characteristics, body shape, voice analysis, and questionnaire responses that were recorded in a previous study [31]. The diagnostic power of the model adapted to classify SCT in the present study is superior to that of QSCCII [33], which has been commonly used for the classification of SCT [34]. Briefly, the facial images of subjects were taken with a digital camera, and several facial points and contours were automatically generated by image processing procedures. Variables for facial points and contours include the following: width, height, areas, angle, depth, and ratio of face shape, forehead, eye, upper eyelid, and noses. For the body shape analysis, eight circumferences such as forehead circumference, neck circumference, axillary circumference, chest circumference, rib circumference, waist circumference, pelvic circumference, and hip circumference, height, weight, and body mass index (BMI) were used to express body shape characteristics. A voice analysis was made using two voice analysis programs, Hidden Markov Model Toolkit (HTK) and Praat. A voice signal having the minimum duration of 40 ms was employed for feature extraction. More than two hundred features from the vowels and the sentences were extracted as an initial set, and eighty-eight features were finally selected for final diagnostic model after applying a genetic algorithm-based feature selection technique. The questionnaire for SCTs includes personality characteristics, which consisted of fifteen questions and can specify general temperaments, eating habits (e.g., whether having regular meals, frequency of eating meals a day, and eating speed), and physiological symptoms (e.g., perspiration, excrement, discomfort in the body, discomfort place when you are unwell, and existence of fatigue).

### 2.3. Definition of Hypertension and DM

Hypertension was defined as a systolic/diastolic blood pressure ≥140/90 mmHg or a patient who was using antihypertensive medications [35]. Diabetes mellitus was defined as high concentrations of fasting glucose ≥100 mg/dL or use of antihyperglycemic agents due to elevated glucose [33].

### 2.4. Health Examination, Questionnaire on Lifestyle, Sleep-Related Factors, and Biochemical Measurements

Comprehensive health examinations and questionnaire-based interviews were conducted during every follow-up visit. The BMI was calculated as the weight in kilograms divided by height in meters squared measured to the nearest 0.1 cm or 0.1 kg. Waist circumference (cm) was measured at the narrowest point between the lower rib and the iliac crest and then repeated for two more measurements to calculate the average value. The arterial BP was measured noninvasively in a sitting position with a mercury sphygmomanometer on the nondominant arm. 

The questionnaire included information on lifestyle and sleep-related factors such as smoking status (never, former, or current), alcohol consumption (gram/day), exercise (30 min/2 times/week), the frequency of a habitual snorer (more than 4 times/week), the presence of daytime nap and excessive daytime sleepiness, efficient sleep (yes/no), sleep duration/day, and excessive daytime sleepiness score. The biochemical analyses for plasma fasting glucose and insulin, serum total cholesterol, triglycerides, and HDL cholesterol were performed in the Seoul Clinical Laboratories (Seoul, Republic of Korea).

### 2.5. Portable Sleep Measurements

An unattended home polysomnogram was performed using an Embla portable sleep monitoring system (Embletta X100, Embla, USA), which consisted of one channel electroencephalogram (EEG) (C4-A1), electrooculogram (EOG), chin electromyogram (EMG), a pressure transducer air flow (PTAF) sensor, thoracic and abdominal respiratory movements sensor, EKG, and pulse oximetry. According to the American Academy of Sleep Medicine (AASM) scoring manual for respiratory events [36], Apneas are defined as decrements in airflow of at least 90% from a previous baseline for a period of 10 seconds. Hypopneas are defined as a decrease in oronasal flow of ≥30% with a corresponding decrease in oxygen saturation on pulse oximetry of ≥4%. The AHI is defined as the number of apneas and hypopneas per hour of total sleep time (TST). The oxygen desaturation index (ODI) is defined as the frequency of decrease in oxygen saturation on pulse oximetry of ≥4% per hour of total sleep time. The presence of OSA was determined if an AHI of ≥5 had been reported. 

### 2.6. Statistical Analysis

Data are expressed as a mean ± SD. The significant differences of the means were evaluated using one-way ANOVA for continuous variables and *χ* ^2^ test for categorical data. In addition, we conducted a multiple logistic regression analysis to estimate an odds ratio (OR) of the presence of OSA in relation to different SCTs with a 95% confidence interval (CI). The potential confounding variables included in the multivariate models for sleep apnea and SCTs were age, sex, BMI, current smoker, alcohol consumption, exercise, and the presence of hypertension and diabetes. Statistical analysis was performed with SAS version 9.1. All *P* values <0.05 were considered as significant. 

## 3. Results

### 3.1. General Characteristic of Participants

The 652 participants (male: 339 and female: 313) who underwent a portable PSG were included for the analysis. The general characteristics of the participants according to the SCT are summarized in Table 1. The TE type was older, heavier, and had a higher proportion of males than those of the other types. The TE type consumed about twice as much alcohol as the SE and SY types (*P* = 0.0091). In the blood chemical analysis, the levels of fasting glucose, fasting insulin, TG, HDL cholesterol were significantly higher in the TE type as compared to any other groups. In addition, the TE group exhibited a higher SBP and DBP level and a higher prevalence of hypertension and DM, as compared to the SE and the SY type. 

### 3.2. The Association between the SCT and Sleep-Related Variables

We investigated the association between the SCT and sleep-related variables (Table 2). In a one-way ANOVA analysis, the frequency of a habitual snorer, sleep efficiency, siesta, and excessive daytime sleepiness did not differ significantly among the SCTs, but AHI and ODI, which represent severity of OSA, were significantly higher in the TE type than those of the SE and the SY type (*P* < 0.0001, both).

### 3.3. Odds Ratios for OSA according to the SCT

In order to estimate the odds ratios for OSA in relation to the SCT, we conducted a logistic regression analysis (Table 3). When considering the SE type as a reference, the odds ratios for OSA were 6.26 (95% CI, 3.40–11.54; *P* < 0.0001) in the TE type and 2.55 (95% CI, 1.34–4.86; *P* = 0.0043) in the SY type in the univariate model. Even after adjusting for confounding factors such as age, sex, BMI, the presence of hypertension and DM, current smoking, alcohol consumption, and exercise, the TE type still had a 2.34-fold (95% CI, 1.11–4.94; *P* = 0.0262) increased risk for OSA, whereas the SY type had not show increasing risk. 

## 4. Discussion

This study aimed to explore the association between SCT and OSA in a large population in Korea. We found that the TE type is an independent risk factor for OSA. Moreover, we confirmed the previous results that the TE type is more obese and has an increased level of SBP and DBP and a prevalence of DM and hypertension in the TE type is higher than those of the SY and SE types. 

The reason why the TE type had a higher likelihood of having OSA is unknown, but it could be partially explained by different craniofacial structures in terms of the pathophysiology of OSA. It has been recognized that a structural factor (such as obesity and craniofacial morphology) and functional factors (including upper airway dilator muscle activity, ventilator control stability, and arousal threshold), individually or synergistically contribute to the development of OSA [37–39]. These factors could act on upper airway patency and ventilation control system to develop or maintain OSA during sleep. Among them, especially in obesity and abnormalities in craniofacial morphology, can play a very significant role in the development of OSA [40]. It has been known that they account for approximately two-thirds of the variation in AHI [41]. 

It has been demonstrated that many patients with OSA are obese, and conversely, patients who are overweight have a high prevalence of OSA. It has also been reported that reducing body weight by approximately 10% decreases the AHI up to 26% [42]. Obesity may result in upper airway dysfunction *via* the neurohormonal system [43] and cause mechanical problems in upper airway and chest wall movement which result in reduced lung volume by excess accumulation of fat around the upper airway passage and chest wall, respectively [44]. 

Abnormal anatomic structures including skeletal factors such as a short maxilla and mandible length, retropositioning of them, and soft tissue features such as an enlarged tongue, soft palate, uvula, and large parapharyngeal fat pads, may influence the caliber of the upper airway [40]. Such abnormalities in craniofacial structure may contribute to the development of OSA by narrowing the upper airway and increasing airway resistance during sleep directly or *via* synergistic interaction with obesity. It seems that obesity and craniofacial anatomy contribute differentially to the development of OSA across ethnic groups. The interethnic comparison study between Chinese and Caucasian reported that the craniofacial structure and BMI contribute differentially to OSA. Specifically, Chinese who exhibited more severe anatomical restriction than Caucasian with higher BMI had more severe OSA. This result could imply that the development of OSA is influenced more by craniofacial structure as compared to obesity in the Asian population [45]. According to Yu's study which examined the cephalometric features in obese (BMI ≥ 27) and nonobese patients (BMI < 27) with OSA syndrome using a cephalometric analysis [46], the anteroposterior distance of the bony nasopharynx and oropharynx was shorter than those of obese patients. Moreover, in the stepwise regression analysis, the narrowing of the oropharynx and inferior displacement of the hyoid bone were revealed as critical factors for AHI in nonobese subjects, suggesting that the craniofacial bony structures may be important predisposing factors for OSA in nonobese patients in the same ethnic groups. It was also reported that the majority of Far East Asian men were nonobese but had severe OSA, who showed significantly decreased cranial base dimensions, when compared to white men [47]. 

Although the possibility of the existence of nonanatomical factors among the SCTs for the development of OSA could not be excluded, we speculate that there will be some kind of difference in craniofacial type structure, in that (1) our study participants had the same ethnicity, that is, Asian, (2) they represented BMI as low as those of the subjects in the aforementioned study, (3) the classification of the Sasang types is in part based on analyzing facial morphology, and (4) with a TE type they still had a high risk for OSA after controlling for BMI.

We conducted multiple logistic regression analyses to evaluate the effect of constitution on OSA after taking into account the confounding variables, including alcohol consumption, BMI, and smoking status, which have been known as risk factors for OSA. Even after the adjustment, the TE type still had a higher risk of having OSA than the SE and SY types, revealing that the TE constitution is an independent risk factor for OSA. 

Given that OSA contributes to an elevation of blood pressure, impaired glucose tolerance, and obesity, it is possible to think that OSA is one of the causes of a high prevalence of hypertension, DM, and MS shown in the TE type as previous studies reported. In a four-year follow-up study, even a mild OSA has approximately increased twice the likelihood of developing a new hypertension compared to patients without OSA (AHI < 5) [42, 48, 49]. Another study that investigated the relationship between OSA and MS also has revealed that the presence of OSA, which is defined by AHI ≥ 5, had five times the risk of having MS [50]. When stratified by AHI, conversely, more than half of the patients with mild OSA had MS. Thus, we suggest that the higher likelihood of having OSA and the level of AHI in the TE type may account for abnormal BP regulation and blood lipid profiles and a high prevalence of DM, hypertension, and MS, which has been consistently reported in the TE type. 

## 5. Conclusion

In conclusion, the TE constitutional type is an independent risk factor for the OSA. We suggest that craniofacial differences may exist among the individual SCTs. Further studies will be needed to elucidate factors mainly affecting on the development and severity of OSA among the SCTs. 

## Figures and Tables

**Table 1:** General characteristics of participants according to Sasang constitution types.

Variable	TE	SE	SY	*P* value
Number of cases	364	82	206	
Age	58.17 ± 7.41^1^	55.04 ± 5.55	56.33 ± 6.46	0.0001
Female, *n* (%)	141 (38.7)	47 (57.0)	125 (60.7)	<0.0001
Smoking, *n* (%)				
Never	185 (50.8)	56 (68.3)	138 (67.0)	<0.001
Former	124 (34.1)	15 (18.3)	46 (22.3)	
Current	55 (15.1)	11 (13.4)	22 (10.7)	
Exercise^2^, *n* (%)	146 (40.1)	34 (41.5)	95 (46.1)	0.37
Alcohol consumption (g/day)	10.74 ± 19.75	5.42 ± 27.58	5.75 ± 19.05	<0.01
BMI (kg/m^2^)	26.48 ± 2.19	21.69 ± 1.79	23.20 ± 1.93	<0.0001
WHR (waist-to-hip ratio)	0.91 ± 0.05	0.84 ± 0.06	0.85 ± 0.06	<0.0001
Fasting glucose (mg/dL)	105.66 ± 28.40	90.98 ± 11.67	94.28 ± 14.67	<0.0001
Fasting insulin (uU/mL)	10.05 ± 9.04	7.28 ± 2.81	7.74 ± 3.26	<0.0001
Total cholesterol (mg/dL)	194.63 ± 34.78	199.96 ± 35.68	201.05 ± 32.76	<0.01
HDL cholesterol (mg/dL)	45.5 ± 10.8	53.4 ± 15.8	53.0 ± 15.1	<0.0001
SBP (mmHg)	118.27 ± 13.68	109.71 ± 13.73	110.95 ± 16.16	<0.0001
DBP (mmHg)	78.08 ± 9.66	72.73 ± 8.71	72.82 ± 9.99	<0.0001
HTN, *n* (%)	187 (51.4)	16 (19.5)	52 (25.2)	<0.0001
DM, *n* (%)	122 (33.5)	12 (14.6)	47 (22.8)	<0.001

Abbreviations: BMI: body mass index; HDL: high-density lipoprotein; SBP: systolic blood pressure; DBP: diastolic blood pressure; HTN: hypertension; DM: diabetes mellitus.

^1^Values are mean ± SD.

^2^30 min/2 times/week.

**Table 2:** The association between sleep variables and Sasang constitution types.

Variable	TE	SE	SY	*P* value
Number of cases	364	82	206	
Habitual snorer, *n* (%)	91 (24.3)	13 (15.7)	41 (19.1)	0.13
Efficient sleep, yes (%)	59 (15.7)	17 (20.2)	34 (15.8)	0.57
Siesta, *n* (%)	145 (38.5)	29 (34.5)	72 (33.5)	0.45
EDS, *n* (%)	22 (6.0)	7 (8.5)	21 (10.2)	0.19
Sleep duration, h	6.9 ± 1.2	6.6 ± 1.1	6.8 ± 1.1	0.26
ESS	5.5 ± 3.2	5.9 ± 3.1	5.4 ± 3.4	0.53
AHI	8.9 ± 9.6*	3.9 ± 5.9	5.0 ± 6.0	<0.0001
ODI	8.4 ± 9.1*	3.1 ± 4.7	4.2 ± 5.2	<0.0001

Abbreviations: EDS: excessive daytime sleepiness; ESS: Epworth sleepiness scale; AHI: apnea-hypopnea index; ODI: oxygen desaturation index.

*P < 0.0001 versus the rest groups.

**Table 3:** Odds ratio of OSA in relation to Sasang constitution types.

Type	Number of OSA	Odds ratio (95% CI) of OSA by the Sasang types			
		Crude	Model 1^a^	Model 2^b^	Model 3^c^
SE	14	Reference	Reference	Reference	Reference
SY	71	2.55 (1.34–4.86)**	2.45 (1.27–4.72)**	1.92 (0.98–3.76)	1.94 (0.99–3.80)
TE	159	6.26 (3.40–11.54)***	4.89 (2.62–9.13)***	2.32 (1.10–4.89)*	2.34 (1.11–4.94)*

^a^Data are a adjusted for age and sex.

^b^Data are djusted for age, sex, BMI, hypertension, and DM.

^c^Data are a djusted for age, sex, BMI, hypertension, DM, current smoker, alcohol consumption (g/day), and exercise (30 min/2 times/week).

*P < 0.05.

**P < 0.01.

***P < 0.0001.
